# Psychobiological Stress Regulation in Depressive Women Achieved Through Group Music Therapy: Results From the Randomised‐Controlled Music Therapy for Depression Study

**DOI:** 10.1002/smi.70026

**Published:** 2025-03-22

**Authors:** Christine Gaebel, Marc N. Jarczok, Corina Aguilar‐Raab, Sabine Rittner, Marco Warth, Martin Stoffel, Beate Ditzen

**Affiliations:** ^1^ Institute of Medical Psychology Center for Psychosocial Medicine Heidelberg University Hospital Heidelberg Germany; ^2^ Ruprecht Karl University Heidelberg Heidelberg Germany; ^3^ Clinic for Psychosomatic Medicine and Psychotherapy University Hospital Ulm Ulm Germany; ^4^ School of Social Sciences University of Mannheim Mannheim Germany; ^5^ School of Therapeutic Sciences SRH University Heidelberg Heidelberg Germany

**Keywords:** cortisol, depression, heart rate variability, music therapy, randomised controlled trial, stress

## Abstract

Major depressive disorder (MDD) is a stress‐related disease that affects women more often than men. Music therapy (MT) has been shown to be effective in the treatment of MDD. However, clinical trials investigating the effects of MT on psychological and psychobiological stress‐related outcomes in women suffering from MDD are still scarce. This study was conducted as a randomised controlled trial, with participants assigned to either an intervention group (IG), which received group MT (GMT), or a waitlist control group (CG), which received GMT 6 months later. The primary objective was to assess the impact of GMT on psychological stress outcomes (chronic stress, stress coping, and stress experienced in daily life) and psychobiological stress markers (diurnal salivary cortisol levels and circadian heart rate variability), considering the effects of both group allocation and time. Outcome measurements were taken before, immediately after, and—for some variables—10 weeks following the intervention period. A total of 102 women 18–65 years old and diagnosed with current MDD took part in the study. Overall, the IG demonstrated significantly stronger stress‐reducing effects than the CG. Significant improvements were observed in general stress coping, positive thinking, daily life stress, and cortisol levels. GMT is a cost‐effective and non‐invasive approach to effectively address the stress‐related psychological and psychobiological burden associated with MDD. To demonstrate long‐term effects and gain a better understanding of the underlying mechanisms, further methodologically robust studies are needed.

**Trail Registration:**

The MUSED study was pre‐registered at the German Clinical Trials Registry (DRKS00016616). All study‐related procedures were published in detail in a study protocol.

## 1 Background

Major depressive disorder (MDD), a leading cause of disability worldwide, disproportionately affects women (Liu et al. 2024; World Health Organization 2024) and is intricately linked with stress (Hammen 2015). As the global burden of depression continues to rise, there is an urgent need for effective, accessible interventions that address both depressive symptoms (DS) and their associated stress‐related mechanisms, particularly for women. In this randomised controlled trial (RCT), we investigated the potential of group music therapy (GMT) as a novel approach to managing MDD in women through its presumed effects on subjective and psychobiological stress‐related outcomes.

### 1.1 Depression and Stress

The relationship between stress and MDD is well‐established (Weinmann et al. 2025), with stress often both a precursor and a symptom of depressive episodes (Hammen 2015). Stress in MDD manifests at various levels. On the psychological level, MDD is associated with increased chronic stress (Hussenoeder et al. 2022) and increased experiencing of stress in daily life (Feng et al. 2023), as well as maladaptive stress coping (Orzechowska et al. 2022). Prolonged exposure to stress can also lead to dysregulation of the hypothalamic‐pituitary‐adrenal (HPA; Rein et al. 2019) axis and autonomic nervous system (ANS; Kim et al. 2018; Valenza 2023). The associated imbalances can manifest as alterations in diurnal cortisol secretion (Adam et al. 2017; Dedovic and Ngiam 2015; Doane et al. 2013; Stetler and Miller 2011) and heart rate variability (HRV) patterns (Choi and Jeon 2020; Jarczok et al. 2018, 2017; Wu et al. 2023). These changes can create a viscous circle leading to increased susceptibility to stress and impaired emotion regulation while contributing to the development and maintenance of DS (Porges 1997; Thayer and Lane 2000). Simultaneously, this creates opportunities to integrate therapies for MDD at both psychological and physical levels.

### 1.2 Music Therapy

Music therapy (MT) is defined as ‘the systematic use of music within a therapeutic relationship that aims to restore, maintain and promote emotional, physical, and mental health’ (German Society of Music Therapy 2023, para. 1). MT is an approach capable of addressing both stress (Witte et al. 2022) and MDD symptoms (Aalbers et al. 2017; Leubner and Hinterberger 2017; Wang et al. 2024; Zhao et al. 2016). However, the existing body of research is insufficient, and the underlying mechanisms of action remain largely unexplained.

#### 1.2.1 Music Therapy and Stress

MT has emerged as a promising approach to reduce stress on both a self‐report and a psychobiological level (Witte et al. 2022). Studies indicating positive impacts of structured MT interventions on psychological stress specifically showed improvements in stress coping, reduction in anxiety symptoms, and enhancement of overall well‐being (Fancourt et al. 2014; Witte et al. 2022). The positive effects of music on stress recovery have also been studied and published, although the evidence is not conclusively established (Adiasto et al. 2022). The stress‐reducing effect of MT is attributed to various mechanisms. The positive effects of music have been demonstrated via for example emotional regulation through musical expression, promotion of relaxation and mindfulness, distraction from stressors, and stimulation of positive neurochemical processes in the brain (Koelsch 2015). MT in group settings offers the additional benefit of social support, which can further contribute to stress reduction. Participants often report a sense of connectedness and of being in a safe space for emotional expression (Schneidman et al. 2024).

Beyond psychological impacts, MT also shows measurable effects on physiological stress systems (Thoma et al. 2013), for example the sympathetic‐adreno‐medullary (SAM) axis, the HPA, the immune system, and the ANS (Ellis and Thayer 2010; Koehler et al. 2022; Xiao et al. 2023). Several studies suggest that MT can lower heart rate (HR) and increase measures of HR variability (HRV), indicating improved autonomic regulation (Bradt et al. 2013; Raglio et al. 2015). Some research has shown that MT can lead to altered secretion of salivary cortisol (sCort) levels during the day in different settings, suggesting changes in the physiological stress respective relaxation response (Fancourt et al. 2014; Linnemann et al. 2015). However, other studies showed inconsistent findings, suggesting that effects on sCort and HRV may depend on variables such as age and underlying disease (Bradt et al. 2013; Koehler et al. 2022). Also, these opposing findings underpin that results regarding psychobiological markers are not always consistent and effects have been shown in both directions (Gaebel et al. 2023). This is exacerbated by the fact that many publications in MT do not differentiate between the distinct outcome measures, instead referring to them in general terms as *HRV* or *cortisol*.

In summary, MT has shown a promising potential in reducing stress and improving mood, but its specific effects on stress‐related outcomes in women with depression remain understudied. The *Music Therapy for Depression* (MUSED) study examines the impact of a structured GMT intervention on psychological and psychobiological stress‐related outcomes. By assessing both subjective measures and stress‐sensitive biological markers, we aim to provide a comprehensive understanding of how GMT may influence stress‐related outcomes in women with MDD.

### 1.3 Objectives

The MUSED study aimed to evaluate the efficacy of GMT in reducing the symptoms of depression as well as stress‐related outcomes associated with MDD in a female population. We hypothesised that GMT in addition to treatment‐as‐usual (TAU) would lead to an improved stress regulation than TAU alone, as indicated by: (a) reduction in self‐reported chronic stress, (b) reduction in self‐reported daily life stress experience, (c) enhancement of self‐reported stress coping, (d) increased vagally‐mediated HRV indicative of a more adaptive functioning of the ANS, and (e) changes in diurnal pattern of sCort indicative of HPA functionality.

## 2 Methods

### 2.1 Pre‐Registration

The study was preregistered at the German Clinical Trials Registry (DRKS00016616). Detailed procedures were documented in the study protocol (Gaebel et al. 2021). Besides this publication, results focussing on the DS will be reported elsewhere (Gaebel et al. 2025).

### 2.2 Study Design

The MUSED study was a single‐centre, randomised controlled trial (RCT) aimed at investigating the effects of GMT on depression and stress‐related outcomes in daily life in women with MDD. Participants (*N* = 102) were randomly assigned to either the intervention group (IG = GMT plus TAU or the control group) (CG = TAU only). Conducted at Heidelberg University Hospital from August 2019 to May 2021, the study's adherence to protocol, participant safety, and trial integrity were ensured through rigorous monitoring by MF, an independent researcher who was not otherwise involved in the study. All protocol deviations were documented and addressed.

### 2.3 Participants

The procedure for sample size calculation is detailed in the study protocol (Gaebel et al. 2021). Recruitment methods included outreach to local medical institutions, physicians, psychotherapists, and both digital and print media. Eligible participants were females aged 18 to 65 with a diagnosis of current MDD based on the *Structured Clinical Interview for DSM‐V* (SCID‐V‐CV; Beesdo‐Baum et al. 2019). To participate in the study, the criteria for at least mild depression had to be met according to the *Hamilton Depression Rating Scale* (HDRS; Hamilton 1960) and the *Beck Depression Inventory‐II* (BDI‐II; Beck et al. 1996). Exclusion criteria included severe mental disorders and symptoms (schizophrenia, bipolar disorder, borderline personality disorder, substance abuse or addiction, psychotic symptoms, acute suicidality), current participation in another MT, and significant physical illnesses. Neither the type and scope of standard treatment nor the presence of severe physical illnesses were considered exclusion criteria but were collected as control variables. The use of other MT services during the study period was an exclusion criterion.

### 2.4 Randomisation and Blinding

Participants were divided into six cohorts for group allocation, with 16–18 patients per cohort, block‐randomised in a 1:1 ratio into either IG or CG. Randomisation was conducted by IN (see Acknowledgements), an independent collaborator, using the R package blockrand (Snow 2018). After the pre‐assessment and before the start of the intervention phase, participants received sealed envelopes, which they opened themselves and which contained their random allocation. This assured blinding in terms of group allocation both during pre‐assessment. Additionally, the observer ratings during the post‐assessments were conducted in a blinded manner. Blinding of the self‐ratings from the post‐assessment onwards was not possible due to the nature of the intervention. Statistical data analyses was conducted unblinded.

### 2.5 Study Intervention

In this study, the methods and basic attitudes of the provided MT are based on a psychotherapeutic understanding. The semi‐structured MT programme was conducted at the Outpatient Treatment Unit of the Institute of Medical Psychology, Heidelberg University Hospital. It included outpatient therapy groups, each with eight to nine patients, led by two trained music therapists (graduated at the university level) under regular supervision. Sessions were held weekly during evening hours in a spacious, well‐lit room equipped with various musical instruments and arranged with a circle of chairs for patients and therapists. Each participant underwent a 60‐min individual diagnostic and introductory MT session followed by 10 weekly 120‐min GMT sessions. The therapeutic approach incorporated both active and receptive MT techniques, with process‐oriented and partly ritualised interventions selected and timed according to therapists' assessments of patient needs and group dynamics. A diverse array of musical instruments was utilised, including rhythm instruments (e.g., cajon, djembe, small percussion, frame drums) and harmony and melody instruments (e.g., piano, guitar, vibraphone). Additionally, the patients' bodies and voices were integrated through focused interventions for expressive purposes, such as body percussion, singing, and vocal improvisation. Video‐recordings and loggings of therapy sessions were maintained for quality assurance purposes. The therapy sessions were supplemented by specific homework assignments. Adverse events were documented and promptly addressed. EN, an independent researcher (see Acknowledgements), evaluated therapist adherence and consistency across therapy groups throughout the whole intervention phase. More details regarding the GMT programme can be found in the study protocol (Gaebel et al. 2021). TAU comprised the use of psychopharmaceuticals, other psychotherapies, and/or medical interventions.

### 2.6 Data Collection

**FIGURE 1:**
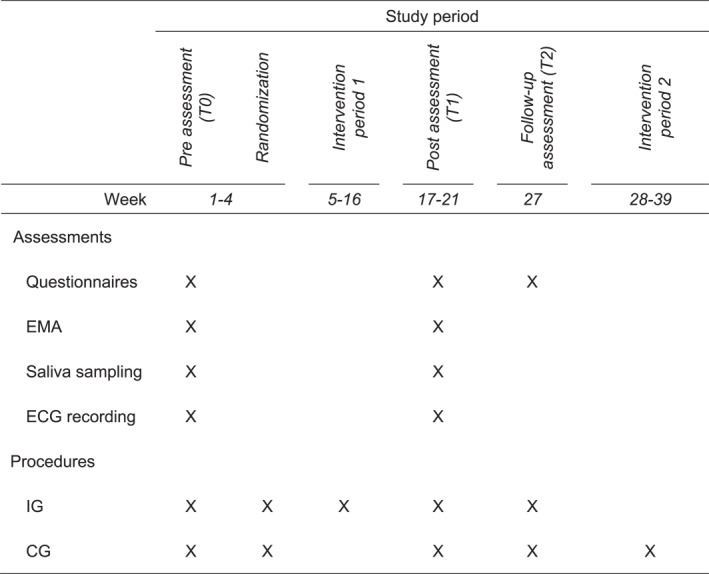
Study procedures. CG = control group; ECG = electrocardiogram; EMA = ecological momentary assessment; IG = intervention group. Questionnaires included the trier inventory of chronic stress and the stress coping inventory. Within the EMA, the national comprehensive cancer network distress thermometer and control variables were assessed. The 24‐h ECG recording, the diurnal saliva sampling, and the accompanied EMA were conducted in parallel on two consecutive days during the participants' daily routine.

The study included pre‐assessments (T0), post‐assessments (T1), and follow‐up assessments (T2). Once informed consent was obtained from participants, we conducted an initial assessment (baseline, T0) comprising self‐ratings and psychobiological assessments accompanied by an ecological momentary assessment (EMA) to determine the participants' current state in their everyday environment. Upon conclusion of the intervention phase, a post‐assessment (T1) was carried out using the same data collection formats as in T0. The follow‐up assessment (T2) occurred 10 weeks after conclusion of the intervention period; it exclusively involved self‐ratings. All self‐report data were assessed online using SoSci Survey Platform. The psychobiological data were collected on two consecutive days at T0 and T1, integrated into the daily life of the participants. An electrocardiogram was used to measure the circadian rhythm of vagally mediated HRV over the course of 48 h, as described by Refinetti et al. (2007). In parallel, six saliva samples per day were taken to determine the moment‐to‐moment sCort pattern. The smartphone‐based EMA captured real‐time perceptions and behaviours within the participants' natural environments, analogous to the sCort assessments. Thus, an event‐related fixed‐occasion design was employed, with the participant's time of awakening serving as the reference event for data collection at six fixed time points throughout the day, according to the recommendations of Adam and Kumari (2009) and Stalder et al. (2016) (T1: awakening, T2: T1 + 30 min, T3: T1 + 45 min, T4: T1 + 150 min, T3: T5 + 480 min, T6: immediately before going to bed). A comprehensive description of the data collection plan for the MUSED study is available in Figure 1 as well as in the study protocol (Gaebel et al. 2021).

### 2.7 Outcome Measures

The GMT programme was conceived and developed as a central component of the MUSED study, the primary objective of which was the amelioration of MDD symptomatology in patients and the mitigation of symptoms associated with MDD. A comprehensive account of all outcome measures gathered during the MUSED study can be found in the study protocol (Gaebel et al. 2021). The present work focuses on the effects of GMT on stress‐regulatory mechanisms that are typically impaired in people suffering from depression.

Self‐reported stress was measured using (a) the *Trier Inventory for Chronic Stress—Short Screening Scale for Chronic Stress* (TICS‐SSCS, 12 items; Schulz and Schlotz 1999), (b) a reduced version of the *Stress Coping Inventory* (SCI, 18 items; Satow 2012) both at T0‐T2, and (c) the *National Comprehensive Cancer Network Distress Thermometer* (single item visual analogue scale; Mehnert et al. 2006) within the EMA at T0‐T1. The psychometric properties of the TICS, SCI, and NCCN Distress Thermometer have been evaluated in several studies. The short version of the TICS demonstrated good internal consistency (*α* = 0.88) and a satisfactory model fit in confirmatory factor analysis (Petrowski et al. 2020, 2019). The SCI showed internal consistency (Cronbach's alpha) ranging from 0.75 to 0.87 across the seven subscales. The factorial validity of the SCI was confirmed through confirmatory factor analysis (Satow 2024). Regarding the NCCN Distress Thermometer, its German version exhibited good discriminative ability, particularly for identifying high distress levels (HADS cut‐off > 11) with Area Under the Curve values between 0.71 and 0.763. Using a cut‐off of 5, the Distress Thermometer showed a sensitivity of up to 84% and specificity of up to 47% for moderate distress (HADS cut‐off > 8) (Labouvie et al. 2023).

Indicators of psychobiological stress regulation were measured at T0‐T1 interpreting (d) momentary sCort measurement, for each day and for each person, indicative of HPA regulation and (e) the circadian heart‐rate patterns *Midline Estimating Statistic of Rhythm* (MESOR) of *Root Mean Square of Successive Differences* (RMSSD), indicative of vagal activity (Laborde et al. 2017).

Control variables and sociodemographic data included age, body mass index (BMI), and childhood trauma as measured by the *Childhood Trauma Questionnaire* (CTQ; 31 items; self‐rating; T0; Bernstein et al. 1994; Klinitzke et al. 2012). Additionally, the EMA included control variables regarding consumption behaviour (such as momentary food and beverage intake), which is known to have an impact on sCort, see Stoffel et al. (2021). The Inter‐Assay‐CV of the sCort data was 8%, the Intra‐Assay‐CV was 3%.

### 2.8 Data Analysis

The MUSED study utilised an intention‐to‐treat (ITT) approach, incorporating all available data (AAD) into the statistical analyses. All statistical analyses were conducted using the R software environment for statistical computing (R Core Team 2024). The datasets used for this paper have been made available on the heiDATA Dataverse Network (https://doi.org/10.11588/data/L9TEVQ). We hypothesised a cross‐level interaction between time and group assignment. Multilevel modelling (MLM) was used for hypothesis testing, accounting for nested data structures, and was performed using the R package *nlme* (Pinheiro et al. 2020) with a maximum likelihood method of estimation. The Type‐I error probability was set at *α* = 0.05. We controlled for alpha‐inflation in multiple testing using the Benjamini‐Hochberg (BH) method and report the adjusted *p*‐values (Benjamini and Hochberg 1995). Missing data were handled by conducting sensitivity analyses to ensure robustness of findings. For the psychobiological data (sCort and HRV), analyses with and without statistical outliers were compared. For the self‐report data (questionnaires), ITT approaches with AAD were compared via a per‐protocol‐analysis (PPA) according to Andrade (2022). As predefined, the PPA dataset included participants who had attended at least six of 11 therapy sessions.

#### 2.8.1 Two‐Level‐Models

To evaluate the effects on chronic stress (TICS), stress coping (SCI), circadian HRV (MESOR), and sCort parameters (AUC_i_, AUC_g_, and slope), we employed two‐level random intercept MLMs, where measurements (level 1) were nested within participants (level 2). In a previous step, trigonometric regression was performed to estimate circadian rhythm parameters of HRV, which were analysed using multivariate regression models (Refinetti et al. 2007). Age was included as a covariate in all models due to the known decline in HRV with increasing age (Garavaglia et al. 2021). Random intercepts for participants were incorporated into all models to account for individual differences in HRV levels.

#### 2.8.2 Three‐Level Models

Moment‐to‐moment sCort and daily life stress (NCCN Distress Thermometer) were analysed using three‐level MLMs, with measurements (level 1; L1) nested within days (level 2; L2), which in turn were treated as nested within individuals (level 3; L3). Analyses were conducted based on scripts published by Stoffel et al. (2021). A detailed description of the model fitting process for moment‐to‐moment sCort can be found in Supporting Information S1: Appendix A.

## 3 Results

**FIGURE 2:**
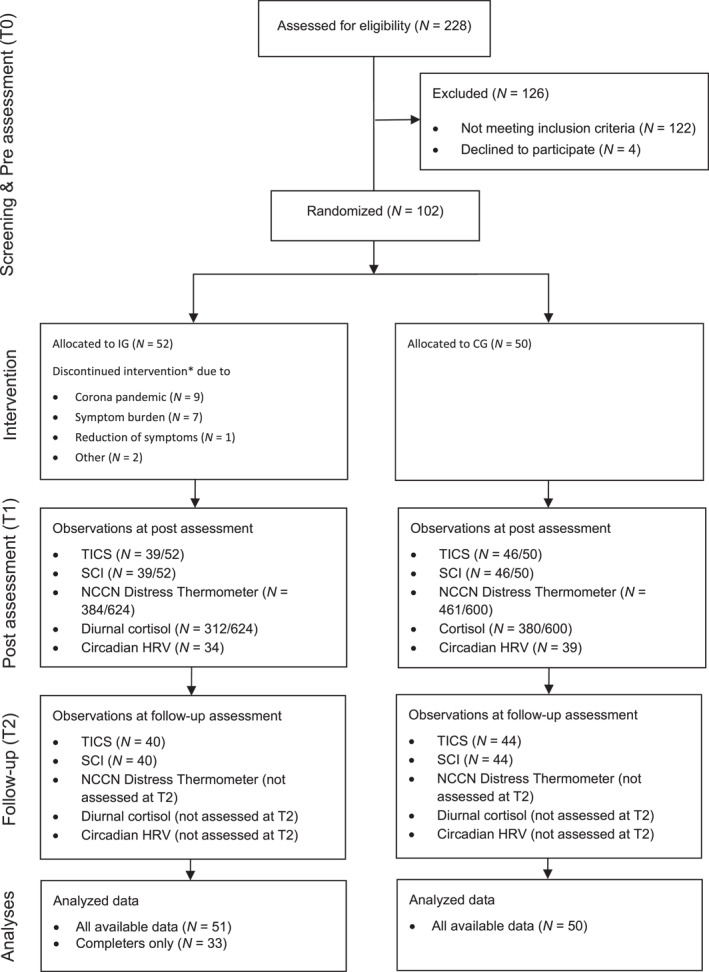
Participant flow chart. Discontinuation of intervention = having taken part in fewer than 6 of the 11 therapy sessions (10 group sessions + anamnestic interview). CG = control group; HRV = heart rate variability; IG = intervention group; NCCN = national comprehensive cancer network; SCI = stress coping inventory; TICS = trier inventory of chronic stress.

A total of 228 potential participants were screened for eligibility. Of these, 102 met the criteria and agreed to join the study. Participants were randomly assigned to either the IG (*N* = 52) or the waiting‐list CG (*N* = 50). The dropout rate from baseline (T0) to the second follow‐up (T2) was 14%. ITT analyses on available data were conducted with a sample of 101 participants. Per‐protocol analyses (PPA) included 83 out of the 102 participants (81.4%): 33 from the IG (63.5%) and all 50 from the CG (100%). Figure 2 provides a detailed overview of the participant flow.

### 3.1 Sample Characteristics

Means and standard deviations of the demographic sample characteristics at baseline (T0) of the outcomes of interest are listed in Table 1. Testing for group differences at T0 did not reveal significance.

**TABLE 1:** Sample and baseline characteristics.

	Total (*N* = 102)			IG (*N* = 52)			CG (*N* = 50)		
Characteristics	*M*	SD	Range	*M*	SD	Range	*M*	SD	Range
General									
Age	46.97	13.06	20–65	47.23	13.61	20–65	46.69	12.59	20–65
BMI	26.19	6.45	16.38–48.77	26.24	6.58	16.38–47.11	26.13	6.38	18.48–48.77
CTQ	54.85	13.85	29–106	56.1	13.57	32–106	53.61	14.15	29–94
BDI‐II	29.61	7.59	13–50	28.25	7.36	14–48	31	7.63	13–50
HDRS 17	19.98	5.17	9–38	19.39	5.18	9–30	20.58	5.14	9–38
Psychological outcomes									
TICS	43.91	7.56	21–60	43.71	6.38	31–54	44.12	8.68	21–60
SCI	46.45	5.79	31–57	46.12	5.46	33–56	46.80	6.14	31–57
Positive thinking	8.48	1.84	4–14	8.45	1.62	5–13	8.51	2.07	4–14
Active stress coping	8.12	1.60	3–12	8.22	1.39	5–12	8.02	1.81	3–12
Social support	8.02	2.25	3–12	7.86	2.23	3–12	8.18	2.29	3–12
Keeping faith	8.61	2.96	4–15	8.71	2.77	4–15	8.51	3.18	4–15
Alcohol and cigarette consumption	13.22	2.74	4–16	12.88	2.74	8–16	13.57	2.73	4–16
NCCN distress thermometer	59.22	14.60	6.5–101	57.76	14.64	6.5–92.58	60.69	14.56	31.09–101
Psychobiological outcomes									
Diurnal cortisol	8.14	2.42	0.78–16.28	8.22	2.61	0.78–16.28	8.07	2.22	3.81–14.06
Circadian HRV (MESOR)	25.5	14.69	6.27–97.77	23.95	10.80	7.52–58.47	27.12	17.88	6.27–97.77

*Note:* This table was created using the *R* package *tableone* (Kazuki Yoshida and Alexander Bartel, 2022).

Abbreviations: BDI‐II = beck depression inventory II (Beck et al. 1996); BMI = body mass index; CG = control group; CTQ = childhood trauma questionnaire (Klinitzke et al. 2012); HDRS = hamilton depression rating scale (Hamilton 1960); HRV = heart rate variability; IG = intervention group; MESOR = midline estimating statistic of rhythm; NCCN = national comprehensive cancer network; SCI = stress coping inventory; TICS = trier inventory of chronic stress.

*p < 0.05, **p < 0.01, ***p < 0.001.

Apropos MDD as a primary diagnosis, the following secondary comorbid mental disorders were also diagnosed: anxiety disorders, post‐traumatic stress disorder, obsessive‐compulsive disorder, attention deficit hyperactivity disorder, and bulimia nervosa. All diagnoses are listed in Table 2.

**TABLE 2:** Diagnoses according to ICD‐10.

	Total (*N* = 102)		IG (*N* = 52)		CG (*N* = 50)	
Diagnoses	*N*	%	*N*	%	*N*	%
Depression diagnoses						
F32.0 (mild, single)	5	4.9	4	7.7	1	2
F32.1 (moderate, single)	16	15.7	7	13.5	9	18
F32.2 (severe, single)	2	3.9	1	1,9	3	6
F33.0 (mild, recurrent)	30	29.4	18	34.6	12	24
F33.1 (moderate, recurrent)	37	36.3	17	32.7	20	40
F33.2 (severe, recurrent)	9	8.8	4	7.7	5	10
F34.1 (dysthymia)	42	41.2	25	48.1	17	34
Comorbidities						
F40.0 (agoraphobia)	6	5.9	3	5.8	3	6
F40.1 (social phobias)	9	8.8	4	7.7	5	10
F40.2 (specific phobias)	2	2	0	0	2	4
F41.0 (panic disorder)	16	15.7	8	15.4	8	16
F41.1 (GAD)	23	22.5	9	17.3	14	28
F42.2 (OCD)	1	1	1	1.9	0	0
F43.1 (PTSD)	18	17.6	9	17.3	9	18
F50.2 (bulimia nervosa)	2	2	1	1.9	1	2
F90.2 (ADHD)	1	1	1	1.9	0	0

*Note:* Assignment of the diagnosis F34.1 in addition to a depressive episode was possible (double depression). This table was created using the *R* package *tableone* (Kazuki Yoshida and Alexander Bartel, 2022).

Abbreviations: ADHD = attention‐deficit hyperactivity disorder; CG = control group; GAD = generalised anxiety disorder; IG = intervention group; OCD = obsessive‐compulsive disorder; PTSD = post‐traumatic stress disorder.

### 3.2 Effects on Self‐Reported Stress

Chronic stress, as measured by the TICS from T0 to T2, decreased more in the IG than in the CG. However, after applying the BH correction, this effect was no longer statistically significant (*b* = −1.9, *p* = 0.056).

Stress coping ability, measured by the SCI from T0 to T2, also improved primarily in the IG, except for the increased *alcohol and cigarette consumption* in both groups. Significant effects were observed for the *positive thinking* subscale (*b* = 0.74, *p* = 0.02) and the total SCI score (*b* = 1.25, *p* = 0.048). On the total SCI score, the IG improved from T0 over to T2 compared to the CG.

Self‐perceived stress in daily life, measured by the NCCN Distress Thermometer from T0 to T1, decreased significantly (*b* = −5.32, *p* = 0.048) more in the IG compared to the CG.

A comprehensive summary of the multilevel model analysis results for the psychological stress outcomes can be found in Supporting Information S2: Appendix B. Supporting Information S3: Appendix C provides the means and standard deviations. Supporting Information S3: Appendix C contains plots of means and standard errors for all outcomes.

### 3.3 Effects on Psychobiological Stress Markers

The fixed effects estimate for the group‐by‐time interaction was significant regarding diurnal levels of moment‐to‐moment sCort (*b* = 0.14, *p* = 0.048; see Supporting Information S4: Appendix D, Figure 2). The interaction indicated that the effects of time (T0 to T1) significantly depended on group assignment, indicating an effect of the intervention on levels of sCort over time. In detail, average sCort levels increased in the IG and remained nearly unchanged in the CG.

For HRV (MESOR), no discernible or statistically measurable group‐by‐time interaction effect was detected (*b* = 0.98, *p* = 0.786; see Supporting Information S4: Appendix D, Figure 2). Both groups hardly differed over time (T0 to T1).

See Supporting Information S5: Appendix E for all multilevel model results, Supporting Information S3: Appendix C for the means and standard deviations, and Supporting Information S4: Appendix D for the plots of means and standard errors of the psychobiological stress outcomes.

### 3.4 Ancillary Analyses

Pearson's correlation coefficient showed no effects between self‐ratings and psychobiological stress outcomes (all *r* < 0.21). Sensitivity analyses of the psychological data comparing the ITT approach with the PPA approach revealed no significant differences except for the TICS. Here, the ITT approach showed a significant effect (*p* = 0.028), which did not remain significant in the PPA analysis (*p* = 0.111). Sensitivity analyses of the psychobiological outcomes showed no differences when comparing datasets with and without outliers.

### 3.5 Side Effects and Therapy Adherence

In total, nine instances of adverse events or side effects were documented throughout the study intervention. This included symptom exacerbation (*n* = 2), somatisation such as unexplained pain and gastrointestinal symptoms (*n* = 2), dissociation (*n* = 2), panic attacks (*n* = 2), and psychotic symptoms (*n* = 1). The adverse effects predominantly occurred when participants were confronting challenging life issues during GMT. The emergence of psychotic symptoms was temporally associated with the patient's prior, unforeseeable substance abuse. The overall adherence to the protocol was high, achieving 92%. This was attributed to complete compliance with the core treatment plan, a 96% rate of conducting anamnestic interviews in presence, and an 81% adherence to the treatment setting.

## 4 Discussion

This study examined the effects of outpatient GMT combined with TAU (IG) versus TAU alone (CG) on self‐report and psychobiological stress‐related outcomes in women suffering from MDD. Overall, the psychological outcomes indicate that GMT consistently exerted greater positive effects on stress, as compared to that of the participants who received TAU alone. Moreover, the IG showed greater reductions of self‐reported stress in daily life despite an increase in the aggregated sCort levels also measured in daily life. No group‐by‐time interaction effects were found for chronic stress, circadian HRV, or the following stress coping strategies: active stress coping, social support, keeping faith, and alcohol and cigarette consumption.

Descriptive examination of the score trajectories reveals that, overall, the IG exhibits more health‐promoting effects on all psychological outcomes than the CG, except for alcohol and cigarette consumption. These findings are consistent with prior research that reports partially beneficial effects of creative arts therapies in general (Martin et al. 2018), and especially of MT to improve stress‐related outcomes (Witte et al. 2022), also in the treatment of MDD (Schäfer 2022). The improvement in stress coping found here through GMT suggests that GMT could also be applied prophylactically during DS‐free intervals in cases of recurrent MDD to reduce the likelihood of relapse.

It is noteworthy that, as typically observed in similar studies, pre‐to‐post effects are detectable, but these effects then tend to plateau or decline, showing only minimal improvement from post to follow‐up (Aalbers et al. 2017; Erkkilä et al. 2021; Witusik and Pietras 2019). Possible reasons for the absence of long‐term effects include: methodological issues (e.g., participant attrition and publication bias), therapy‐related factors (limited duration of therapy, lack of follow‐up), patient‐related factors (compliance, environmental factors, comorbidity, and complexity of mental disorders), and disorder‐specific factors (natural course of depressive episodes, risk of relapse in MDD). For this reason, in the MUSED study therapists assigned homework during the sessions and stressed the importance of applying the new habits to daily life after the therapy. The long‐term effects on psychobiological stress and perceived stress in daily life were not examined in this study, in order to minimise the burden on the already stressed target group and thereby enhance compliance.

Another apparent discrepancy that emerges from the results are the divergent trends of the psychological and the psychobiological outcomes: sCort levels increased in the IG, while self‐perceived stress changed in a health‐promoting way, and HRV remained unchanged in both groups. Since increased stress can be both a cause and a symptom of MDD, the disease is commonly associated with an altered stress response as measured by cortisol levels, for example flattened cortisol levels in stress reactivity, elevated cortisol levels during the recovery phase (Burke et al. 2005), and cortisol slopes flattened from morning to evening (Adam et al. 2017; Doane et al. 2013). Given that cortisol serves to mobilise energy, the observed result of elevated sCort levels in combination with reduced perceived stress could indicate both, either increased stress on a psychobiological level or—in contrast—a rather healthy dynamic of the HPA axis, which is typically fatigued in individuals with MDD. This fatigue of the HPA axis might explain the perceived lack of energy commonly associated with depressive disorders. In the present study, increased sCort levels could have been caused by the fact that the GMT not only consisted of relaxation‐inducing interventions but also predominantly activating elements, which might have resulted in increased HPA activation. Above this, while GMT reduced subjective stress ratings, the intervention might still have increased genuine activity, arousal, and HPA axis activity, as reflected by increased sCort levels.

The absence of a significant group‐by‐time interaction in HRV could be due to the intervention phase being too short to induce a profound change in circadian rhythms at the autonomic level, as has been found in other intervention studies (Goessl et al. 2017; Schumann et al. 2021). Another reason could be that HRV and/or psychological stress and for example well‐being are not related linearly but quadratically, causing the effect to average out (Kogan et al. 2013). Additionally, HRV—similar to other biomarkers—is influenced by many factors, including age, sex, menstrual cycle phase, and physical fitness, making interpersonal comparison of HRV data more challenging. Apart from that, HRV circadian rhythms have not been widely used as an outcome measure in intervention studies (Jarczok et al. 2021, 2019). On the one hand, the high ecological validity is a strength of this outcome measure. However, a drawback is that the measurements were not conducted under laboratory conditions, which may introduce a certain level of artefacts that could potentially distort the results.

### 4.1 Strengths and Limitations

The novelty of this study lies in the combined assessment of the effects of GMT on stress from both a self‐report and a psychobiological perspective within a sufficiently powered randomised controlled study design. The group format of the study intervention may have provided additional benefits through social support and shared experiences among participants. The integration of observational methods with data assessments in real‐life contexts—including biomarkers and an EMA—offered an insightful combination of retrospective self‐reporting and high ecological validity. At the same time, measuring biomarkers in daily life places a substantial burden on the participant. Especially when not measured under laboratory conditions, biomarkers are prone to variability, and the complex interplay of physiological outcomes complicates the interpretation of the results.

The focus on women, with all severity levels of DS, and the homogeneity of the female sample help reduce potential sex‐specific biases, especially in the psychobiological results (Eid et al. 2019).

The provided GMT within an add‐on therapy design offers advantages such as targeted approaches for non‐responders and reduced bias when properly randomised, but it also presents challenges such as increased complexity, potential overestimation of benefits, and limited generalisability (Fava et al. 2003; Gold et al. 2011). The waiting list control group design used here has the disadvantage that expectation effects may have occurred, which could also have had an impact on individual stress. Personal feedback from study participants also suggests that dropouts occurred immediately after randomisation in the CG because the study participants were not willing to wait the required 6 months to receive treatment. Notably, the GMT applied here was not primarily designed as a stress intervention but conceptualised to alleviate the DS. However, as stress is an important trigger for depressive episodes and MDD and also a consequence of prolonged depression, the focus on stress might provide some crucial information on GMT effects.

The COVID‐19 pandemic introduced challenges such as increased attrition rates and data loss, although steps were taken to mitigate these issues, including recruiting additional participants and addressing pandemic‐related concerns during therapy sessions. It cannot be excluded that the challenging conditions under which the therapy sessions took place during the COVID‐19 pandemic impacted the participants' stress perception and physical responses. Furthermore, there is limited evidence on manualized MT for MDD treatment, which underscores the need for a structured approach to the GMT programme. Although this approach reflects clinical practice, it makes it difficult to pinpoint specific working factors. Efforts are ongoing to develop a flexible therapy manual that can be adapted to various contexts.

Side effects during GMT were similar to those reported in other psychotherapy studies, indicating comparable effects (Moritz et al. 2019). However, the generalisability and reproducibility of the findings are limited due to the sample characteristics and the partially manualized intervention.

Upcoming studies should include larger and more diverse samples to enable subgroup analyses by sex and account for confounding factors, such as trauma history and the type of TAU used. Additionally, it would be advisable to incorporate a combination of different physiological measures beyond the stress‐related biomarkers examined here, such as interleukin‐6, which is indicative of inflammatory responses (Ting et al. 2020), alpha‐amylase, which reflects ANS activity (Pallich et al. 2022), as well as possible epigenetic markers (Schaumburg et al. 2020) and brain imaging techniques (Pilmeyer et al. 2022). This would provide a more comprehensive understanding of how MT impacts the complex interplay of various interconnected physical stress systems. If feasible, daily life measurement of biomarkers could be complemented by immediate assessments before, during and after therapy sessions to better understand MT's effects on individuals with MDD and the underlying mechanisms involved. Implementing active CGs is recommended to reduce bias from expectation effects and to avoid overestimating treatment efficacy (Baxter and Allmark 2013). To get a better understanding of the long‐term effects of MT, future studies should examine the long‐term effects on psychobiological stress outcomes and perceived stress in daily life. Although the therapy duration in the MUSED study (11 sessions in total 21 h) exceeded the 16 h identified as most effective by Gold et al. (2009), extending the sessions might make the intervention's effectiveness more sustainable. It is also advisable to collect side effect data in the CG in future research.

## 5 Conclusions

The MUSED study further underscores previous findings that GMT has the immediate potential to promote health by positively influencing both psychological and psychobiological stress. As such, it represents a non‐pharmacological, cost‐effective addition to the field of stress prevention or reduction. However, more research with robust study designs and intervention durations longer than 10 weeks are needed to understand how the health‐promoting effects of GMT can be sustained.

## Author Contributions

C.G., C.A.‐R., M.N.J., M.S., and B.D. were responsible for conceptualising the study. The study intervention was conducted by C.G., and S.R. Data collection was carried out by C.G. and M.W. Statistical analyses were performed by C.G., M.S., M.N.J., and M.W. C.G. and M.S. prepared the initial draft of the manuscript. All authors contributed equally to critically revising the manuscript, and they provided final approval for the version to be published.

## Ethics Statement

The study was conducted according to the required standards of the Declaration of Helsinki. Ethical approval was obtained from the local ethics committee of Heidelberg Medical Faculty (S‐545/2016).

## Consent

The collection, processing and use of data in the MUSED study were based on the legal basis of voluntary and informed consent of the participants (according to §§ 4, 4a BDSG). The consent to participate was obtained before study entry and could be withdrawn at any time and without justification.

## Conflicts of Interest

The authors declare no conflicts of interest.

## Permission to Reproduce Material From Other Sources

The authors have nothing to report.

## Supporting information

Supporting Information S1

Supporting Information S2

Supporting Information S3

Supporting Information S4

Supporting Information S5

## Data Availability

All study data will be stored in the heiDATA Dataverse Network. They are accessible via: (https://doi.org/10.11588/data/L9TEVQ) in pseudonymised form.
