# Supplementary material for: Psychobiological Stress Regulation in Depressive Women Achieved Through Group Music Therapy: Results From the Randomised‐Controlled Music Therapy for Depression Study
Source: Stress Health. 2025 Mar 22;41(2):e70026. doi: 10.1002/smi.70026 (PMC11929563; doi:10.1002/smi.70026)
Supplement: Supplementary file 1 — Supporting Information S1 [file SMI-41-e70026-s003.docx]

**Appendix A: Description of the model fitting process of moment-to-moment diurnal salivary cortisol**

SCort values measured during the first 25 minutes after awakening were removed from the data analysis in order to exclude the effect of the cortisol awakening response, which could have biased the data. To enable normally distributed residuals of the MLMs, sCort was log-transformed prior to all analyses. Outliers beyond three standard deviations of the respective mean (i.e., depending on the level of analyses, this could be deviations from a mean for each measurement occasion, for each day, or for all persons) were flagged for later sensitivity analyses. Potential confounders known to alter sCort levels (see Stoffel et al., 2021) were included as covariates (see Appendix C, Table 1); they were centered on the grand mean. To control for time within days (and the circadian rhythm of sCort), time in minutes relative to awakening was included (where time within days at awakening was assumed to be zero). Potential polynomial time trends within days (fixed effects; e.g. quadratic time trends) were tested by comparing models with different time trends against each other. Further, potential random effects on L2 and L3 were tested separately by comparing the baseline model (including all fixed effects for covariates and time within days, as well as random intercepts on L2 and L3) against a new model that was built the same way as the baseline model but extended by one additional random effect at a time. However, after the process was completed, all model assumptions according to Pinheiro, J.C., Bates, D.M., DebRoy, S., Sarkar, D., R. Core Team (2020) were met.

In case the random effect significantly improved the model fit, it was later entered into the final model. Model comparisons were performed by using likelihood-ratio tests as well as the Bayesian Information Criterion (BIC). To fit the final model, the group-by-time interaction was entered into the model with all random and fixed effects. However, this full model (including all relevant random effects as discovered in the approach described above, with a total of 4 variances and 6 covariances on L1 and on L2) did not converge. Given the importance of random effects for the estimation of accurate fixed effects in confirmatory hypothesis testing (Barr et al., 2013), we decided to keep random effects in the final model and to simplify the random effects structure by dropping the correlations between random effects (see Barr et al., 2013 for details) using the pdDiag()-function of the *nlme* package (Pinheiro, J.C., Bates, D.M., DebRoy, S., Sarkar, D., R. Core Team, 2020). As a result, the final model converged. Distributional assumptions of the MLM residuals were tested according to standard procedures as published by Pinheiro, J.C., Bates, D.M., DebRoy, S., Sarkar, D., R. Core Team (2020) and Stoffel et al. (2021). In this process, the residuals on L1 were found to not be independently distributed. Thus, for the final model, we specified an autocorrelation structure of order 1 with a continuous covariate (time between occasions in two-hour intervals) using the corCAR1()-function of the *nlme* package, which significantly improved the model fit. All further model assumptions were met. Thus, the final model reported here is built using a diagonal random-effects variance–covariance matrix and includes the autocorrelation structure (both described above).
