# Supplementary material for: Psychobiological Stress Regulation in Depressive Women Achieved Through Group Music Therapy: Results From the Randomised‐Controlled Music Therapy for Depression Study
Source: Stress Health. 2025 Mar 22;41(2):e70026. doi: 10.1002/smi.70026 (PMC11929563; doi:10.1002/smi.70026)
Supplement: Supplementary file 2 — Supporting Information S2 [file SMI-41-e70026-s004.docx]

**Appendix B: Results of the psychological stress outcomes**

*Table 1: TICS levels as a function of time-by-group interactions*

| Fixed effects | | | | |  |
| --- | --- | --- | --- | --- | --- |
| *Predictors* |  | *Estimates* | *SE* | *p* |  |
| Fixed effects |  |  |  |  |  |
| Intercept |  | 43.40 ^***^ | 1.06 | **< .001** |  |
| Time |  | -0.89 | 0.59 | **.133** |  |
| Group |  | -0.42 | 1.50 | .779 |  |
| Time*group |  | -1.90 | 0.86 | .056^a^ |  |
| Random effects (SD) | | | | | |
| Level 2 (across participants) | | | | |  |
| Intercept |  | 5.7 |  |  |  |
| Time |  | 1.47 |  |  |  |
| Residual |  | 5.21 |  |  |  |
| *N* |  |  |  |  |  |
| Participants |  | 100 |  |  |  |
| Observations |  | 269 |  |  |  |

*Note.* ^a^ p-values pertaining to the focal predictors (time*group interactions) were adjusted with the Benjamini-Hochberg correction (Benjamini and Hochberg, 1995). The bold print is significant as following: ** p<0.05   ** p<0.01   *** p<0.001*. Groups = intervention group vs. control group; Time = T0-T2. TICS = Trier Inventory of Chronic Stress; SE = standard error; SD = standard deviation.

*Table 2: SCI levels as a function of time-by-group interactions*

|  | | Fixed effects | | | | | | | | | | | | | | | | | | | | | | |
| --- | --- | --- | --- | --- | --- | --- | --- | --- | --- | --- | --- | --- | --- | --- | --- | --- | --- | --- | --- | --- | --- | --- | --- | --- |
| Subscales |  | Positive thinking | | |  | Active stress coping | | |  | Social support | | |  | Keeping faith | | |  | Alcohol and cigarette consumption | | |  | Total | | |
| *Predictors* |  | *Estimates* | *SE* | *p* |  | *Estimates* | *SE* | *p* |  | *Estimates* | *SE* | *p* |  | *Estimates* | *SE* | *p* |  | *Estimates* | *SE* | *p* |  | *Estimates* | *SE* | *p* |
| Intercept |  | 8.59 *** | 0.27 | **< .001** |  | 8.05 ^***^ | 0.22 | **< .001** |  | 8.18 ^***^ | 0.32 | **< .001** |  | 8.54 ^***^ | 0.42 | **< .001** |  | 13.48 ^***^ | 0.39 | **< .001** |  | 46.84 ^***^ | 0.82 | **< .001** |
| Time |  | -0.07 | 0.16 | **.656** |  | 0.35 ^**^ | 0.12 | **.003** |  | 0.07 | 0.14 | **.600** |  | 0.04 | 0.13 | **.765** |  | 0.12 | 0.14 | **.376** |  | 0.55 | 0.35 | **.117** |
| Group |  | 0.05 | 0.37 | **.891** |  | 0.30 | 0.32 | **.342** |  | -0.21 | 0.44 | **.641** |  | 0.19 | 0.59 | **.748** |  | -0.56 | 0.55 | **.313** |  | -0.21 | 1.15 | **.852** |
| Time*group |  | 0.74 ^*^ | 0.23 | **.02 ^a^** |  | -0.06 | 0.17 | **.786** ^a^ |  | 0.28 | 0.20 | **.244** ^a^ |  | 0.05 | 0.18 | **.786** ^a^ |  | 0.31 | 0.20 | **.2** ^a^ |  | 1.25 ^*^ | 0.51 | **.048 ^a^** |
|  | Random effects (*SD*) | | | | | | | | | | | | | | | | | | | | | | | |
| Level 2 (across participants) | | | | | | | | | | | | | | | | | | | | | | | | |
| Intercept |  | 1.52 |  |  |  | 1.21 |  |  |  | 2.01 |  |  |  | 2.73 |  |  |  | 2.61 |  |  |  | 4.96 |  |  |
| Time |  | 0.68 |  |  |  | 0.02 |  |  |  | 0.64 |  |  |  | 0.23 |  |  |  | 0.69 |  |  |  | 0.82 |  |  |
| Residual |  | 1.18 |  |  |  | 1.09 |  |  |  | 0.99 |  |  |  | 1.16 |  |  |  | 0.89 |  |  |  | 3.09 |  |  |
| *N* |  |  |  |  |  |  |  |  |  |  |  |  |  |  |  |  |  |  |  |  |  |  |  |  |
| Participants |  | 100 |  |  |  | 100 |  |  |  | 100 |  |  |  | 100 |  |  |  | 100 |  |  |  | 100 |  |  |
| Observations |  | 269 |  |  |  | 269 |  |  |  | 269 |  |  |  | 269 |  |  |  | 269 |  |  |  | 269 |  |  |

*Note.* ^a^ p-values pertaining to the focal predictors (time*group interactions) were adjusted with the Benjamini-Hochberg correction (Benjamini and Hochberg, 1995). The bold print is significant as following: ** p<0.05   ** p<0.01   *** p<0.001*. Groups = intervention group vs. control group; Time = T0-T2. SCI = Stress coping inventory; SE = standard error; SD = standard deviation.

*Table 3: NCCN distress thermometer (momentary burden) as a function of a time-by-group interaction*

| Fixed effects | | | | | | |
| --- | --- | --- | --- | --- | --- | --- |
| *Predictors* |  | *Estimates* | *SE* | | *p* | |
| Intercept |  | 62.84 ^***^ | 2.02 | | **< .001** | |
| Time |  | -0.45 | 1.53 | | **.771** | |
| Group |  | -3.19 | 2.74 | | .246 | |
| Time*group |  | -5.32 ^*^ | 2.26 | | **.048** **^a^** | |
| Random effects (*SD*) | | | | | | |
| Level 3 (across participants) | | | | | | |
| Intercept |  | 8.59 |  | | |  |
| Time |  | 1.99 |  | | |  |
| Level 2 (across days) | | | | | | |
| Intercept |  | 16.87 | |  | |  |
| Time |  | 3.16 | |  | |  |
| Residual |  | 13.21 | |  | |  |
| *N* |  |  | |  | |  |
| Participants |  | 100 | |  | |  |
| Observations |  | 1978 | |  | |  |

*Note.* p-values pertaining to the focal predictors (time*group interactions) were adjusted with the Benjamini-Hochberg correction (Benjamini and Hochberg, 1995). The bold print is significant as following: ** p<0.05   ** p<0.01   *** p<0.001*. Groups = intervention group vs. control group; Time = T0-T1. NCCN = National Comprehensive Cancer Network; SE = standard error; SD = standard deviation.
