# Supplementary material for: Psychobiological Stress Regulation in Depressive Women Achieved Through Group Music Therapy: Results From the Randomised‐Controlled Music Therapy for Depression Study
Source: Stress Health. 2025 Mar 22;41(2):e70026. doi: 10.1002/smi.70026 (PMC11929563; doi:10.1002/smi.70026)
Supplement: Supplementary file 3 — Supporting Information S3 [file SMI-41-e70026-s002.docx]

**Appendix C: Means and standard deviations of the psychological and psychobiological outcomes**

|  |  | Pre | | | | | | |  | Post | | | | | | |  | Follow-Up | | | | | | |
| --- | --- | --- | --- | --- | --- | --- | --- | --- | --- | --- | --- | --- | --- | --- | --- | --- | --- | --- | --- | --- | --- | --- | --- | --- |
|  |  | Intervention group | | |  | Control group | | |  | Intervention group | | |  | Control group | | |  | Intervention group | | |  | Control group | | |
| Outcomes |  | *N* | *M* | *SD* |  | *N* | *M* | *SD* |  | *N* | *M* | *SD* |  | *N* | *M* | *SD* |  | *N* | *M* | *SD* |  | *N* | *M* | *SD* |
| Psychological Outcomes |  |  |  |  |  |  |  |  |  |  |  |  |  |  |  |  |  |  |  |  |  |  |  |  |
| TICS (total) |  | 51 | 43.71 | 6.38 |  | 50 | 44.12 | 8.68 |  | 39 | 38.08 | 8.32 |  | 46 | 41.37 | 9.19 |  | 40 | 37.83 | 10.42 |  | 44 | 42.91 | 9.2 |
| SCI (total) |  | 51 | 46.12 | 5.46 |  | 50 | 46.8 | 6.14 |  | 39 | 50.46 | 6.40 |  | 46 | 47.26 | 6.66 |  | 40 | 49.78 | 6.41 |  | 44 | 47.32 | 7 |
| Positive thinking |  | 51 | 8.45 | 1.62 |  | 50 | 8.51 | 2.07 |  | 39 | 9.97 | 2.02 |  | 46 | 8.7 | 2.26 |  | 40 | 9.83 | 2.49 |  | 44 | 8.3 | 2.14 |
| Active stress coping |  | 51 | 8.22 | 1.39 |  | 50 | 8.02 | 1.81 |  | 39 | 9 | 1.52 |  | 46 | 8.41 | 1.88 |  | 40 | 8.78 | 1.48 |  | 44 | 8.66 | 1.68 |
| Social support |  | 51 | 7.86 | 2.23 |  | 50 | 8.18 | 2.29 |  | 39 | 8.85 | 2.01 |  | 46 | 8.15 | 2.21 |  | 40 | 8.58 | 2.11 |  | 44 | 8.3 | 2.38 |
| Keeping faith |  | 51 | 8.71 | 2.77 |  | 50 | 8.51 | 3.18 |  | 39 | 9.1 | 2.69 |  | 46 | 8.59 | 3.26 |  | 40 | 8.93 | 2.68 |  | 44 | 8.25 | 3.28 |
| Alcohol & cigarette consumption |  | 51 | 12.88 | 2.74 |  | 50 | 13.57 | 2.73 |  | 39 | 13.54 | 2.59 |  | 46 | 13.41 | 2.95 |  | 40 | 13.68 | 2.8 |  | 44 | 13.82 | 2.87 |
| NCCN Distress Thermometer |  | 561 **^b^** | 58.22 | 20.92 |  | 572 | 60 | 20.76 |  | 384 | 51.8 | 20.1 |  | 461 | 58.05 | 22.25 |  |  |  |  |  |  |  |  |
| Psychobiological Outcomes |  |  |  |  |  |  |  |  |  |  |  |  |  |  |  |  |  |  |  |  |  |  |  |  |
| sCort |  | 454 **^b^** | 8.22 | 6.23 |  | 453 | 8.07 | 5.79 |  | 312 | 8.48 | 6.24 |  | 380 | 8.04 | 5.72 |  |  |  |  |  |  |  |  |
| Circadian HRV (MESOR) |  | 44 | 23.95 | 10.8 |  | 42 | 27.12 | 17.88 |  | 34 | 23.55 | 11.51 |  | 39 | 26.42 | 16.16 |  |  |  |  |  |  |  |  |

*Note. M* = mean; *SD* = standard deviation; TICS = Trier Inventory of Chronic Stress; SCI = Stress Coping Inventory; NCCN = National Comprehensive Cancer Network; MESOR = Midline estimating statistic of rhythm (calculated according to Refinetti et al., 2007); sCort = (diurnal) salivary cortisol. High values at the TICS indicate high levels of chronic stress. High values at the SCI indicate the use of adaptive stress coping strategies, except the subscale “alcohol and cigarette consumption” (inverse). High values at the NCCN Distress Thermometer indicate high levels of burden/stress. The unit of sCort ist in ng/ml. **^b^** In these cases, *N* indicates the number of observations instead of the number of participants.
