# Supplementary material for: Psychobiological Stress Regulation in Depressive Women Achieved Through Group Music Therapy: Results From the Randomised‐Controlled Music Therapy for Depression Study
Source: Stress Health. 2025 Mar 22;41(2):e70026. doi: 10.1002/smi.70026 (PMC11929563; doi:10.1002/smi.70026)
Supplement: Supplementary file 4 — Supporting Information S4 [file SMI-41-e70026-s005.docx]

**Appendix E: Results of the psychobiological stress outcomes**

*Table 1: Moment-to-moment diurnal salivary cortisol as a function of a time-by-group interaction*

| Fixed effects | | | | | | |  |
| --- | --- | --- | --- | --- | --- | --- | --- |
| *Predictors* |  | *Estimates* | *SE* | *p* | | |  |
| Intercept |  | 2.47 ^***^ | 0.05 | **< .001** | | |  |
| Time (pre/post) |  | -0.08 | 0.04 | **.057** | | |  |
| Time within days (linear) |  | > -0.01 ^***^ | 0.00 | **< .001** | | |  |
| Time within days (quadratic) |  | < 0.01 ^***^ | 0.00 | **< .001** | | |  |
| Group |  | -0.04 | 0.06 | .459 | | |  |
| Time*group |  | 0.14 ^*^ | 0.06 | **.048^a^** | | |  |
| Momentary depression |  | < 0.01 | < 0.01 | .586 | | |  |
| Food |  | > -0.01 | < 0.01 | .204 | | |  |
| Beverage |  | > -0.01 ^***^ | < 0.01 | **< .001** | | |  |
| Activity |  | < 0.01 | < 0.01 | .501 | | |  |
| Caffeine |  | -0.02 | 0.03 | .568 | | |  |
| Sleep quality |  | > -0.01 | < 0.01 | .710 | | |  |
| Sleep problems |  | 0.02 | 0.04 | .660 | | |  |
| Age |  | < 0.01 | < 0.01 | .082 | | |  |
| BMI |  | -0.01 | < 0.01 | .096 | | |  |
| Contraceptives |  | 0.05 | 0.08 | .530 | | |  |
| Random effects (*SD*) | | | | | | |  |
| Level 3 (across participants) | | | | | | |  |
| Intercept |  | 0.21 |  | | |  | |
| Time within days (linear) |  | < 0.01 |  | | |  | |
| Level 2 (across days) | | | | | | |  |
| Intercept |  | 0.04 |  | |  | |  |
| Time within days (linear) |  | 8.63 |  | |  | |  |
| Time within days (quadratic) |  | 1.54 |  | |  | |  |
| Food |  | 6.73 |  | |  | |  |
| Beverage |  | < 0.01 |  | |  | |  |
| Residual |  | 0.41 |  | |  | |  |
| *N* |  |  |  | |  | |  |
| Participants |  | 95 |  | |  | |  |
| Observations |  | 1382 |  | |  | |  |

*Note.* ^a^ p-values pertaining to the focal predictors (time*group interactions) were adjusted with the Benjamini-Hochberg correction (Benjamini and Hochberg, 1995). The bold print is significant as following: ** p<0.05   ** p<0.01   *** p<0.001*. Groups = intervention group vs. control group; Time = T0-T1. SE = standard error; SD = standard deviation.

*Table 2: HRV (MESOR) levels as a function of time-by-group interactions*

| Fixed effects | | | | |  |
| --- | --- | --- | --- | --- | --- |
| *Predictors* |  | *Estimates* | *SE* | *p* |  |
| Fixed effects |  |  |  |  |  |
| Intercept |  | 27.11 ^***^ | 2.08 | **< .001** |  |
| Time |  | -1.01 | 2.16 | **.641** |  |
| Group |  | -2.91 | 2.92 | .322 |  |
| Age |  | -0.41*** | 0.10 | **< .001** |  |
| Time*group |  | 0.98 | 3.11 | .786^a^ |  |
| Random effects (SD) | | | | | |
| Level 2 (across participants) | | | | |  |
| Intercept |  | 12.82 |  |  |  |
| Time |  | 11.29 |  |  |  |
| Residual |  | 4.82 |  |  |  |
| *N* |  |  |  |  |  |
| Participants |  | 94 |  |  |  |
| Observations |  | 159 |  |  |  |

*Note.* ^a^ p-values pertaining to the focal predictors (time*group interactions) were adjusted with the Benjamini-Hochberg correction (Benjamini and Hochberg, 1995). The bold print is significant as following: ** p<0.05   ** p<0.01   *** p<0.001*. Groups = intervention group vs. control group; Time = T0-T1. HRV = Heart rate variability; MESOR = Midline estimating statistic of rhythm (calculated according to Refinetti et al., 2007); SE = standard error; SD = standard deviation.
