# Supplementary material for: Psychobiological Stress Regulation in Depressive Women Achieved Through Group Music Therapy: Results From the Randomised‐Controlled Music Therapy for Depression Study
Source: Stress Health. 2025 Mar 22;41(2):e70026. doi: 10.1002/smi.70026 (PMC11929563; doi:10.1002/smi.70026)
Supplement: Supplementary file 5 — Supporting Information S5 [file SMI-41-e70026-s001.docx]

**Appendix D: Plotted means and standard errors of psychological and psychobiological outcomes**


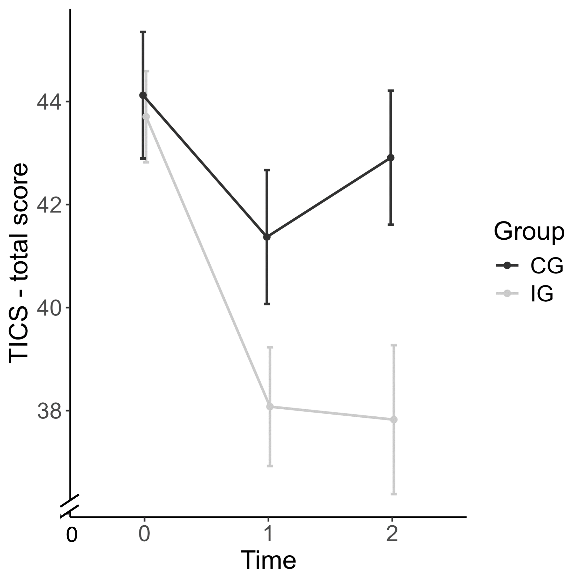

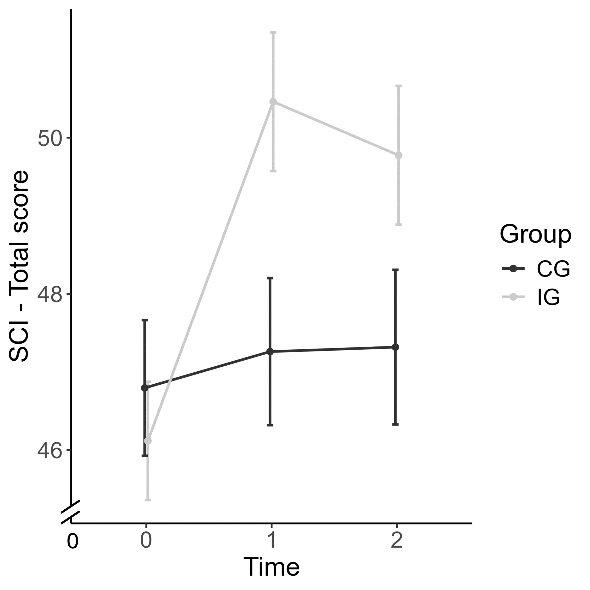
*Figure 1: Plotted means and standard errors of the psychological stress outcomes.*


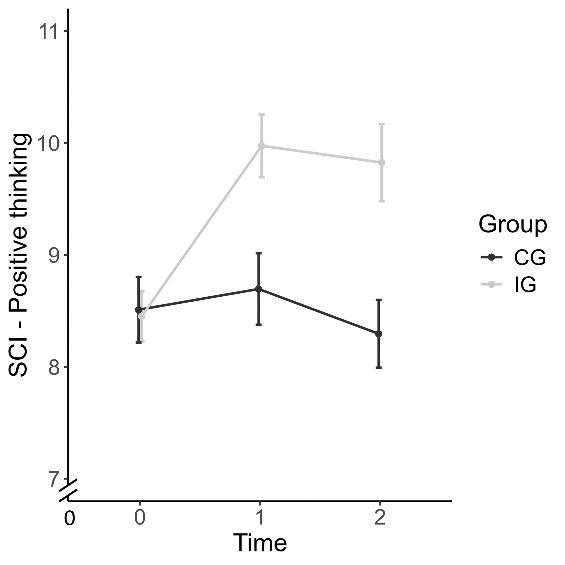

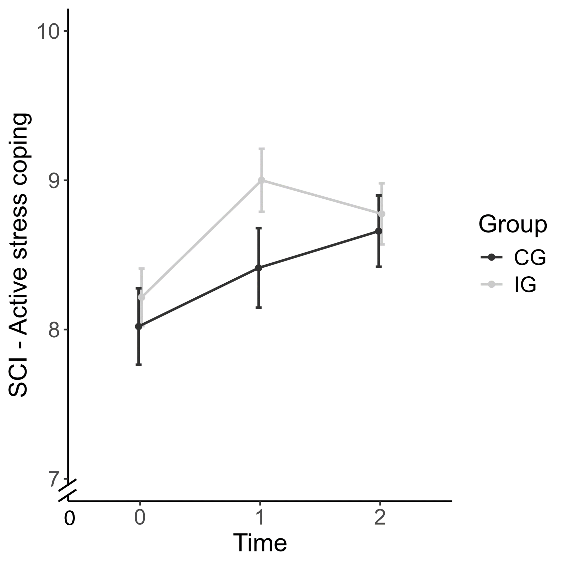


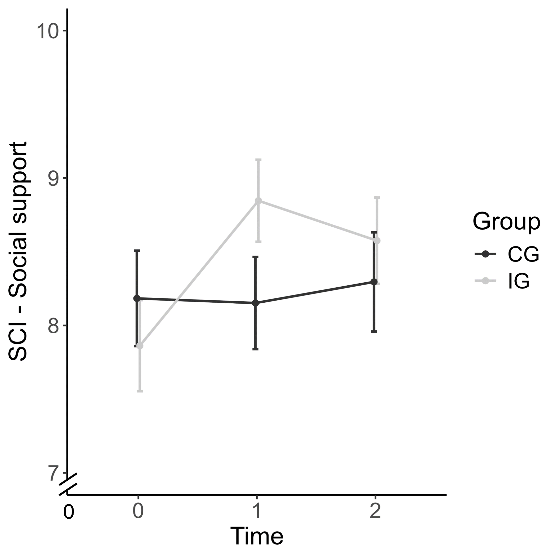

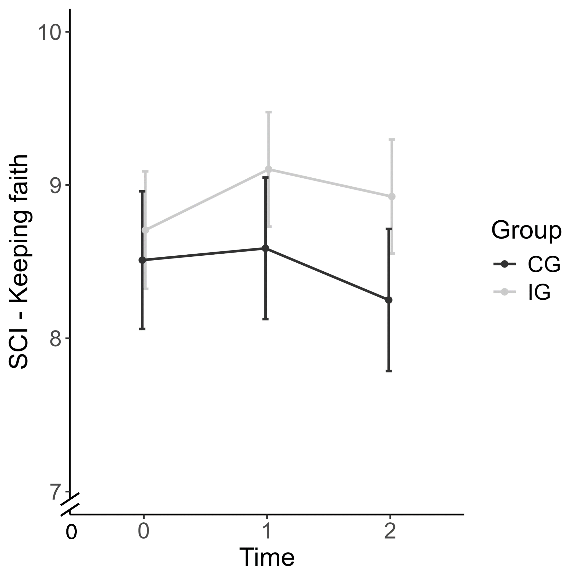

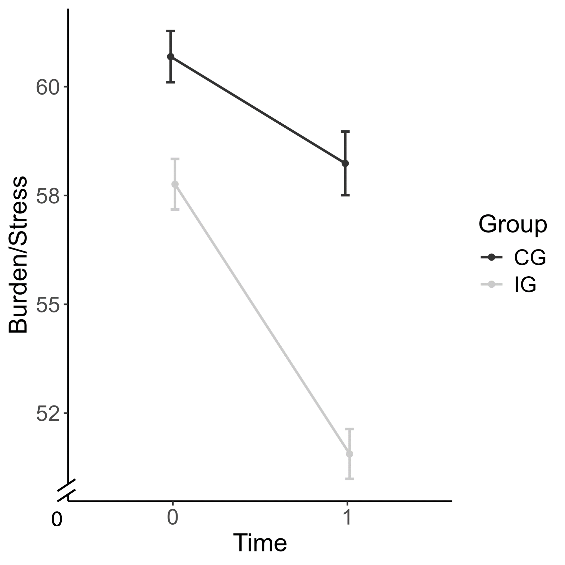


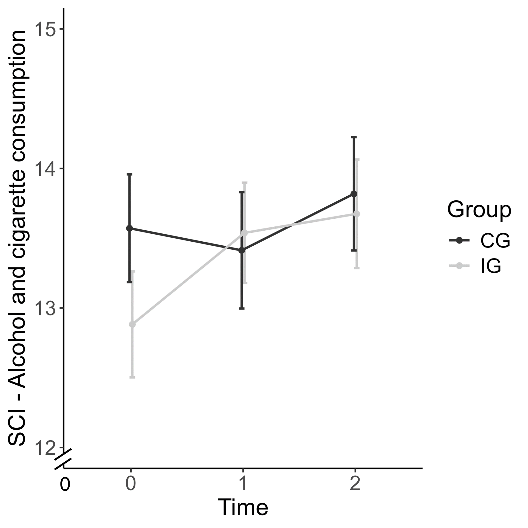


*Note.* SCI = Stress coping inventory; TICS = Trier inventory of chronic stress; CG = control group; IG = intervention group. Time: 0 = pre, 1 = post, 2 = follow-up. Error bars indicate standard errors. High values at the TICS indicate high levels of chronic stress. High values at the SCI indicate the use of adaptive stress coping strategies, except the subscale “alcohol and cigarette consumption” (inverse). High values at the NCCN Distress Thermometer indicate high levels of burden/stress.

*Figure 2: Plotted means and standard errors of psychobiological outcomes (diurnal cortisol and circadian HRV/MESOR).*


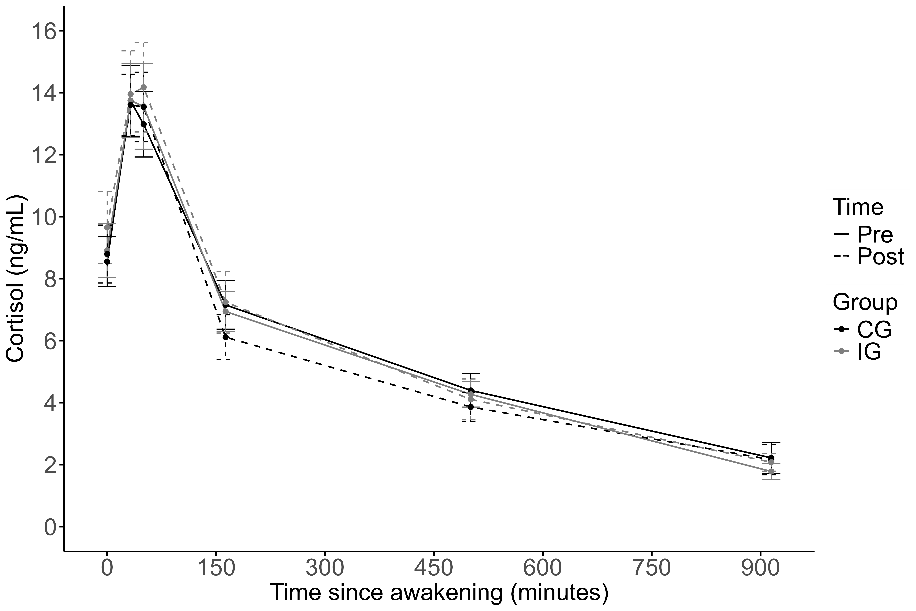


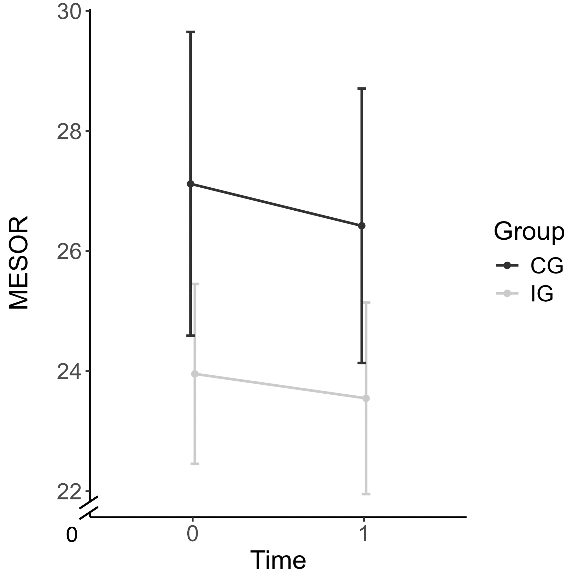


*Note.* CG = control group; IG = intervention group; MESOR = Midline estimating statistic of rhythm (calculated according to Refinetti et al., 2007). Time in the left figure indicates minutes since awakening; time in the right figure: 0 = pre, 1 = post. error bars indicate 95% confidence intervals.
